# Eye-Tracking-Based Interventions for School-Age Specific Learning Disorders: A Narrative Review of Functional Assessment and Gaze-Contingent Training

**DOI:** 10.3390/jemr19030042

**Published:** 2026-04-24

**Authors:** Pierluigi Diotaiuti, Francesco Di Siena, Salvatore Vitiello, Alessandra Zanon, Pio Alfredo Di Tore, Stefania Mancone

**Affiliations:** Department of Human Sciences, Society and Health, University of Cassino and Southern Lazio, 03043 Cassino, Italy; francesco.disiena@unicas.it (F.D.S.); salvatore.vitiello@unicas.it (S.V.); a.zanon@unicas.it (A.Z.); pioalfredo.ditore@unicas.it (P.A.D.T.); s.mancone@unicas.it (S.M.)

**Keywords:** eye tracking, specific learning disorders, functional assessment, oculomotor assessment, gaze-contingent training, dyslexia, dysgraphia, dyscalculia

## Abstract

Eye tracking (ET) provides process-level indices of how students sample task-relevant information during core academic activities. In school-age learners (6–18 years) with specific learning disorders (SLDs; dyslexia, dysgraphia, and dyscalculia), ET can complement behavioural assessment by quantifying oculomotor patterns linked to decoding, model–production coordination, and stepwise strategy execution. This narrative review synthesises ET findings in SLD across reading, handwriting/copying, and arithmetic and translates them into an applied framework for school-oriented use. We summarise key metrics and Areas of Interest (AOI)-based analyses, highlight technical and data-quality requirements for valid acquisition in educational settings, and outline compact functional assessment protocols integrated with standard academic and neuropsychological measures. Building on these foundations, we propose six hypothesis-driven gaze-contingent paradigms (H1–H6) as preliminary models for future experimental testing rather than as established interventions, and we map each to its current level of empirical support, specifying primary gaze outcomes and curriculum-relevant behavioural endpoints. We emphasise that eye-movement findings in specific learning disorders are heterogeneous and may vary as a function of age, task demands, and comorbidity. Accordingly, credible training effects require retention and transfer probes under standard, non-contingent display conditions, appropriate controls, and explicit developmental interpretation. Eye tracking is positioned as complementary functional evidence and as a platform for experimentally testable, mechanism-based interventions in school-age specific learning disorders.

## 1. Introduction

Specific learning disorders (SLDs) are neurodevelopmental conditions characterized by persistent difficulties in the acquisition and use of academic skills, such as reading, writing, and mathematics, despite adequate intelligence and educational opportunities. Dyslexia and dyscalculia represent the most prevalent forms of SLDs and are associated with long-term academic and psychosocial consequences when not identified early [1,2]. Conventional diagnostic approaches primarily rely on standardized behavioural assessments, which provide valuable outcome measures but offer limited insight into the cognitive and perceptual processes underlying learning difficulties [3].

Eye tracking (ET) has increasingly been adopted as a complementary methodology for investigating learning-related cognitive processes. By providing continuous, high-temporal-resolution recordings of eye movements, ET allows direct observation of how visual attention is allocated during task execution. Applied to reading and learning tasks, ET has proven particularly informative for distinguishing between efficient and inefficient processing strategies [4,5,6].

Beyond its descriptive value, ET can also be used to modify the task environment in real time. In gaze-contingent paradigms, stimulus presentation or feedback is dynamically adapted as a function of where and for how long a participant looks. These approaches can serve two complementary goals: supporting attention allocation during task execution and implementing training loops that reinforce efficient visual strategies.

To clarify how these paradigms may be translated into educational settings, gaze-contingent interventions commonly rely on three operational principles. First, the system can highlight or enhance task-relevant information only when gaze reaches the relevant area (e.g., progressive cueing), thereby promoting systematic sampling of critical elements. Second, the system can reduce the salience of irrelevant regions (e.g., masking or attenuation outside a window around gaze) to discourage distractor-driven scanning. Third, gaze can be used to trigger immediate feedback or pacing rules (e.g., advancing stimuli only after sufficient fixation), shaping temporal regularity and sustained engagement.

Although gaze-contingent approaches have been explored in several clinical and experimental domains, including training programs targeting atypical gaze patterns and oculomotor control [7,8,9,10], their application to school-age specific learning disorders still lacks a clear, practice-oriented synthesis. In the present review, we use this framework to organise and discuss SLD-oriented intervention hypotheses and protocol components, focusing on reading-, handwriting-, and arithmetic-related tasks and their measurable eye-movement outcomes.

This mechanism-based perspective is especially relevant to reading. Influential models of eye-movement control link fixation duration and regressions to lexical and post-lexical processing demands and to attentional allocation across foveal and parafoveal information [4,5,6,9,11]. In SLD, slower or less stable eye-movement patterns are therefore interpreted primarily as markers of inefficient decoding and integration rather than as isolated oculomotor deficits [12,13,14].

A second interpretive caution concerns heterogeneity and comorbidity. Specific learning disorders are not behaviourally or cognitively uniform, and eye-movement findings may vary across subtypes, severity levels, linguistic context, task structure, and the presence of co-occurring attentional or executive-control difficulties. For this reason, we do not assume that a single gaze profile characterises all learners with specific learning disorders. Rather, we treat eye tracking as a process-tracing method that may help identify different functional pathways to poor performance.

This review adopts a mechanism-based view: gaze-contingent supports are expected to be useful only insofar as they reduce task-irrelevant visual search and help the learner allocate attention to the information required by the academic operation (grapheme–phoneme mapping during reading, model–production checking during handwriting, and stepwise inspection/alignment during arithmetic) [4,5,6,11,15,16].

This position is also consistent with the broader independent review literature on ET in dyslexia assessment and gaze-contingent training in related clinical populations (see Table 1). Accordingly, intervention effectiveness should be evaluated through converging evidence: curriculum-relevant behavioural outcomes and eye-movement changes consistent with the hypothesised mechanism, plus retention and transfer beyond the training display.

We use ET not as a generic “visual” measure but as a process-tracing method: Eye movements index moment-by-moment allocation of attention to task-relevant information, and gaze-contingent rules can selectively support or shape this allocation when it constitutes a bottleneck for academic performance. This implies clear domain constraints. In reading, the relevant bottleneck concerns mapping and integration at the word and line level; in arithmetic, it concerns encoding of operands and operators and stepwise strategy execution with visuospatial alignment; in attention-related learning difficulties, it concerns sustained goal maintenance and resistance to distractor-driven scanning. Accordingly, ET-based interventions are warranted only when the hypothesised deficit can be operationalised as measurable off-task gaze dispersion or inefficient sampling within task-defined Areas of Interest (AOIs) and when behavioural outcomes demonstrate retention and transfer beyond the training display [4,5,6,15,16]. Here and throughout this manuscript, AOIs refer to predefined task-relevant regions within the visual display or workspace used to quantify where and for how long gaze is allocated.

This intervention-oriented perspective is grounded in a growing body of evidence indicating that children with SLDs exhibit systematic alterations in eye-movement behaviour, including longer fixation durations, shorter saccades, and increased regression rates during reading and problem-solving tasks. These patterns are commonly interpreted as reflecting inefficiencies in decoding, integration, and executive control rather than purely oculomotor deficits [12,13,14]. Beyond gaze position, pupillary responses have also been shown to provide reliable information about cognitive load and mental effort during learning activities, offering an additional window into attentional engagement [17,18,19].

At the same time, the interpretation and practical use of these findings remain constrained by important methodological limitations. Much of the existing ET literature is based on laboratory-bound, screen-mounted systems requiring rigid head stabilization, which may reduce ecological validity, particularly in pediatric and educational settings [4,5]. There is ongoing debate regarding how eye-movement measures should be interpreted and integrated into functional assessment and intervention practice for SLDs [6].

Previous reviews have addressed only parts of this landscape. For example, recent systematic work has examined the use of eye-tracking technology for dyslexia assessment and diagnosis in school-aged children, with emphasis on hardware, software, and screening-related metrics rather than on functional educational use or intervention design [20]. In parallel, broader reviews of gaze-contingent eye-tracking training have synthesised intervention evidence across mixed neurodevelopmental, psychiatric, and neurological conditions, but not with a specific focus on school-age specific learning disorders or on curriculum-embedded academic tasks such as reading, handwriting, and arithmetic [21]. Accordingly, what remains insufficiently covered is an integrated, practice-oriented synthesis that connects eye-movement measures, cognitive interpretation, technical requirements, functional assessment, and gaze-contingent intervention hypotheses within the specific context of school-age SLD.

Throughout this review, “school-age” refers to children and adolescents in compulsory schooling (approximately 6–18 years). Where primary studies report age or grade, we explicitly indicate it to support the interpretation and transferability of the proposed protocols. This age range should be interpreted cautiously. The label “school-age” is useful for framing the review, but it covers developmental stages that differ substantially in reading automatization, handwriting fluency, executive control, and mathematical strategy use. Consequently, the same eye-movement metric may not have the same functional meaning in a 6-year-old beginner reader and a 16-year-old adolescent. Throughout this review, we therefore treat age and grade as interpretive variables rather than as background descriptors only.

To better position the contribution of the present review, Table 1 summarizes the main review articles currently available in this area and compares their scope, strengths, and limitations. This overview helps clarify what has already been covered in the literature and, importantly, which aspects remain insufficiently addressed. In particular, while previous reviews have focused either on eye tracking for dyslexia assessment or on gaze-contingent interventions across broader clinical populations, no prior synthesis has specifically integrated functional assessment, cognitively interpretable eye-movement measures, technical requirements, and school-oriented gaze-contingent intervention design across reading, handwriting, and arithmetic in school-age specific learning disorders.

**Table 1:** Position of the present review relative to prior reviews.

Review	Main Scope	What It Covers	What It Does Not Cover	Relevance to the Present Review
Toki (2024) [20]	Systematic review of gaze-contingent eye-tracking training in brain disorders	Gaze-contingent training paradigms across neurodevelopmental, psychiatric, and neurological conditions	Not specific to school-age SLD; no structured synthesis of reading, handwriting, and arithmetic tasks; limited educational applicability	Shows the broader intervention background but does not address SLD-specific school-based protocols
Carelli et al. (2022) [21]	Systematic review of eye-tracking technology in dyslexia diagnosis	Dyslexia screening/diagnosis, hardware/software characteristics, diagnostic metrics in school-aged children	Does not cover dysgraphia or dyscalculia; does not focus on functional assessment integration or gaze-contingent intervention design	Shows the diagnostic review background but not the assessment-to-intervention bridge
Present review	Narrative review of ET in school-age SLD	Eye-movement measures, cognitive interpretation, technical requirements, functional assessment, and gaze-contingent training proposals across reading, handwriting, and arithmetic	—	Addresses the applied gap left open by prior reviews

Against this background, the present narrative review aims to provide an applied synthesis of eye tracking in school-age specific learning disorders by integrating four elements: (1) cognitively interpretable eye-movement measures, (2) technical requirements affecting data quality and ecological validity, (3) functional assessment protocols linked to academic tasks, and (4) preliminary hypothesis-driven gaze-contingent paradigms for future testing in reading, handwriting, and arithmetic. In doing so, this review seeks to distinguish clearly between evidence-based findings and intervention proposals that remain experimental.

## 2. Review Methodology

This article was developed as a narrative review with an applied focus on eye-tracking use in school-age specific learning disorders. To improve transparency, we explicitly defined the literature search and selection approach used to identify the studies and review contributions discussed in this manuscript.

### 2.1. Search Strategy

The literature search was conducted in PubMed, Scopus, Web of Science, PsycINFO, and Google Scholar. The empirical literature discussed in the manuscript spans publications from 1982 to 2025, although the studies most directly relevant to school-age SLD and ET-based applied protocols are concentrated in the period 2014–2025. Search terms combined concepts related to eye tracking, specific learning disorders, and intervention or assessment contexts. Representative search strings included combinations such as the following: (‘eye tracking’ OR ‘eye-tracking’) AND (‘specific learning disorder’ OR dyslexia OR dysgraphia OR dyscalculia) AND (assessment OR intervention OR training OR gaze-contingent). Additional hand-searching of reference lists from relevant articles and reviews was also performed to identify further eligible records. The searches were conducted iteratively and updated until January 2026. Database searching was supplemented by backward reference checking of relevant empirical papers and review articles. Because terminology varies across the literature, both “eye tracking” and “eye-tracking” were used during the search phase, together with disorder-specific and task-specific terms. Search results were screened with priority given to studies that could inform functional interpretation, assessment design, or the feasibility of gaze-contingent support in school-oriented tasks.

### 2.2. Eligibility Criteria

We prioritised peer-reviewed articles written in English that were directly relevant to school-age populations with specific learning disorders or closely related academic-task contexts. We included empirical studies using eye tracking in reading, handwriting/writing, or arithmetic-related tasks; studies addressing gaze-contingent or eye-movement-informed intervention approaches; and relevant review papers used to position the present contribution within the existing literature. We excluded studies not focused on school-age participants or not clearly relevant to learning-related tasks; papers dealing exclusively with unrelated ophthalmological or neurological conditions without educational relevance; and publications lacking sufficient methodological detail for interpretive use in the present review. In line with the applied aims of the review, we also retained a limited number of studies from adjacent child populations or task domains when they provided conceptually relevant information about eye-movement-sensitive change, attentional control, or intervention reporting. These studies were not treated as direct evidence for intervention efficacy in specific learning disorders but as contextual material used to inform interpretation and future study design.

### 2.3. Study Selection and Synthesis Approach

Titles and abstracts were screened for relevance, followed by full-text examination of potentially eligible records. The selection process was purposive rather than algorithmic, consistent with the narrative and applied scope of the review. Studies were prioritised when they contributed to at least one of the following four dimensions: interpretation of eye-movement measures in school-age specific learning disorders; technical or quality-control issues affecting data validity; functional assessment during reading, handwriting, or arithmetic tasks; gaze-contingent or eye-movement-informed support paradigms relevant to future intervention design.

No formal risk-of-bias tool or PRISMA-style flow diagram was applied because the aim was not to produce a systematic review or meta-analysis. Rather, this review sought to organise the literature into an applied conceptual framework while making the search logic and study-selection principles explicit. Accordingly, the synthesis should be interpreted as structured narrative evidence mapping rather than as a quantitative estimate of effect size or treatment efficacy.

Representative search strings used to identify the literature relevant to the present review are summarized in Table 2. The strings were designed to capture studies on eye- tracking in school-age specific learning disorders across assessment, functional interpretation, and intervention-oriented applications. 

### 2.4. Methodological Scope and Limitations

This review should be read as a narrative synthesis with methodological transparency, not as a comprehensive systematic review. The included literature is heterogeneous with respect to populations, devices, tasks, gaze metrics, and outcome definitions. As a result, the purpose of the review is to clarify interpretive and applied issues, identify promising mechanisms, and specify what future controlled studies should report, rather than to establish pooled estimates of intervention efficacy.

## 3. Eye-Movement Measures Relevant for School-Age SLD

### 3.1. Eye Movements as Indicators of Learning-Related Cognitive Processes

ET techniques provide a powerful methodological approach for investigating visual attention and cognitive processing during learning activities. In the field of SLDs, eye-movement measures have been widely employed to characterize atypical visual exploration patterns during reading, writing, and numerical tasks, offering insights beyond traditional behavioural indices [7,8].

Core eye-movement metrics include fixations, saccades, regressions, scanpaths, and pupil dynamics. Fixations correspond to periods of relative ocular stability during which detailed visual processing occurs. Fixation duration, frequency, and spatial distribution are sensitive to processing difficulty and attentional allocation. Empirical studies consistently show that children with SLDs exhibit longer and more variable fixations, reduced saccade amplitudes, and increased regression rates compared to typically developing peers, particularly as task demands increase [15,16,22].

Saccades, defined as rapid eye movements between fixations, index the efficiency of visual exploration and oculomotor planning. Altered saccadic amplitudes and irregular sequencing have been reported in dyslexia and dyscalculia and are associated with slower reading speed and reduced comprehension accuracy [9,10]. Regressions, i.e., backward saccades, are especially informative, as elevated regression rates often signal breakdowns in comprehension or monitoring processes [9,12].

Scanpaths describe the temporal sequence of fixations and saccades and provide a global representation of task-related visual strategies. Efficient learners typically display structured, goal-oriented scanpaths, whereas children with SLDs often show fragmented or disorganized patterns, suggesting difficulties in maintaining task goals and integrating visual information over time [13].

Importantly, eye-movement anomalies in dyslexia and other SLDs are heterogeneous. Beyond reading tasks, several studies report altered performance in cognitively demanding oculomotor paradigms (e.g., increased error rates in antisaccade tasks and reduced predictive saccade learning), consistent with difficulties in volitional inhibition and attentional disengagement [23,24]. Other work points to binocular coordination and vergence instability during reading, and systematic reviews suggest that binocular control may diverge from typical development in at least a subgroup of children with dyslexia [25,26]. Classic work also cautions that eye-movement differences can, in some cases, reflect downstream expressions of broader language/phonological difficulties rather than primary ocular causes [27]. Accordingly, throughout this review, we treat eye movements primarily as process-tracing indicators and as an interface for adaptive stimulus presentation. We do not assume that they are the sole causal drivers of learning disorders, and we recommend that intervention studies include both domain outcomes (e.g., standardized reading measures) and mechanistic gaze metrics (including tasks such as rapid automatized naming) to test mediation and subgroup moderation [28].

Developmental variation should also be considered explicitly when interpreting these measures. Fixation durations, saccadic regularity, line-transition control, and task-related scanpath organisation are shaped not only by disorder-related constraints but also by age, reading experience, schooling, and the degree of automatization reached in the relevant academic domain. Therefore, cross-sectional comparisons involving broad “school-age” samples should avoid implying developmental equivalence across grades. Whenever possible, studies should report age- or grade-specific patterns or, at a minimum, discuss how the developmental stage may affect the functional meaning of the observed gaze profile.

### 3.2. Pupillometry and Cognitive Load

In addition to spatial and temporal gaze measures, pupil dynamics provide complementary information about the effort associated with task performance. Variations in pupil diameter reliably reflect changes in attentional demands and processing intensity during cognitive tasks, including reading and problem solving [14,17]. Pupillometry represents a well-established and sensitive index of cognitive load, rather than a coarse or indirect measure [18,19]. Nevertheless, pupillary responses are influenced by several factors, including luminance conditions and emotional arousal, which necessitate careful experimental control and cautious interpretation [14,17]. When these factors are adequately managed, pupillometry provides robust information about sustained attention and mental effort in learning contexts. Importantly, wearable eye trackers increasingly provide automated pupillometry in physical units, enabling the collection of pupil-diameter time series during naturalistic learning activities. This can support applied monitoring of attentional engagement and cognitive load in children, provided that luminance and other confounds are controlled. For example, Neon provides pupillometry streams at high sampling rates and its pupillometry feature has been evaluated in a dedicated test report [29].

### 3.3. AOI and Early Versus Late Eye-Movement Measures

Beyond individual eye-movement events, ET analysis also benefits from spatially structured and temporally differentiated metrics. The use of AOIs constitutes a central analytical strategy in ET research. AOIs enable the quantification of gaze behaviour within predefined regions of visual stimuli, allowing the computation of measures such as first fixation latency, total dwell time, number of visits, and revisits [30]. A critical distinction in ET research concerns early versus late eye-movement measures. Early measures, such as first fixation duration and initial gaze allocation, are thought to reflect early perceptual and attentional orienting processes, whereas late measures, including total dwell time and regressions, index higher-order processes such as integration, monitoring, and compensatory strategies [31].

### 3.4. From Laboratory-Based to Ecologically Valid Eye Tracking

While the measures described above were initially developed in controlled laboratory settings, their educational usefulness increasingly depends on more ecologically valid recording conditions. Traditionally, ET research relied on screen-based systems requiring head stabilization to ensure high spatial accuracy. While these systems have generated valuable insights, their ecological validity is limited, particularly in pediatric and school-based contexts. Recent advances in wearable, head-mounted eye-tracking systems have enabled reliable eye-movement recording without rigid head stabilization, allowing data collection during naturalistic, in situ learning activities. These systems support dynamic mapping of gaze onto real-world educational materials and represent a critical step toward ecologically valid functional assessment of learning-related visual behaviour [32,33].

Recent wearable, head-mounted systems increasingly rely on calibration-free (or minimal-calibration) gaze estimation pipelines, which reduces the need for unnatural head fixation and facilitates in situ assessment in classroom-like activities. For example, the Pupil Labs Neon headset is designed to operate without a user-specific calibration and is intended for naturalistic recordings across environments. A dedicated accuracy evaluation reports a per-subject median gaze-estimation accuracy of 1.8° (at ~1.3 m depth) without user calibration, including both lab and “in-the-wild” conditions [22].

### 3.5. Selecting Metrics: Psychological Significance and Practical Recommendations

Selecting eye-movement metrics for school-age SLD should be driven by psychological interpretability and task demands, rather than by the broad range of output measures available in eye-tracking software. The same metric can index different processes depending on stimulus properties (e.g., word frequency, line length, spacing, layout) and the learner’s profile (e.g., comorbid attentional weaknesses). For this reason, we recommend defining a small set of primary metrics that directly test the hypothesised bottleneck for a given task (e.g., unstable left-to-right progression in reading; insufficient model–production checking in copying; premature column switching in arithmetic), complemented by a limited number of secondary metrics that help disambiguate competing interpretations (e.g., strategy variability, dispersion/off-task gaze, or rereading time). To support metric selection in applied school contexts, Table 3 provides a compact task-oriented guide linking common educational contexts (reading, attention during academic tasks, handwriting/copying, and arithmetic) to a limited set of primary and secondary eye-movement measures, together with their main psychological interpretation and key practical constraints. The table is intended as a selection aid rather than a prescriptive checklist, helping readers prioritise metrics that are both theoretically interpretable and technically feasible under school-oriented acquisition conditions.

As shown in Table 3, metric selection should remain tightly aligned with the functional question being asked. In reading, the most informative indicators are those capturing progression stability and repair processes; in handwriting/copying, the key issue is coordination between model inspection and written output; in arithmetic, the focus shifts to stepwise AOI sampling and visuospatial alignment. Across all contexts, the table also highlights a practical principle: when data quality or hardware precision is limited, robust AOI-based indicators should be prioritised over fine-grained oculomotor measures that require higher sampling rates and more stable calibration.

In applied and school-oriented contexts, metric choice should also reflect hardware and data-quality constraints. When calibration precision or sampling rate is limited (as can occur with classroom-friendly or wearable setups), emphasis should shift toward robust, AOI-based measures that tolerate noise (e.g., time-in-AOI, coarse fixation duration, transitions between AOIs, proportion of off-task gaze, usable-sample percentage) rather than fine-grained oculomotor indices that require high temporal resolution (e.g., microsaccades or velocity-based measures). Across tasks, reporting should prioritise robust statistics (median/trimmed means), within-student contrasts (e.g., easier vs. harder passages; early vs. late trials), and interpretable summaries that can be linked to observable performance.

We recommend treating eye-movement changes as mechanism-consistent markers rather than as standalone evidence of learning or diagnosis. Accordingly, any ET-based metric set should be paired with curriculum-relevant behavioural outcomes (accuracy, error types, fluency, comprehension, and rule adherence) and interpreted in light of speed–accuracy trade-offs and task structure. This “primary + secondary” strategy supports both functional assessment and the design of gaze-contingent protocols, because it forces explicit alignment between the hypothesised mechanism, the chosen gaze indicators, and the behavioural outcomes needed to claim retention and transfer.

A further recommendation is to interpret metric choice developmentally. In younger children, broader AOI-based and progression-level measures may be more robust and more interpretable than fine-grained oculomotor indices, whereas in older children and adolescents, more differentiated metrics may become informative for strategy, monitoring, or repair processes. This is particularly important in school-age specific learning disorders, where developmental delay, developmental divergence, and compensatory strategy use may coexist.

## 4. Technical Requirements and Quality Control

### 4.1. Hardware and Sampling Rate

Once the relevant eye-movement measures have been identified, their practical interpretation depends on adequate technical acquisition conditions. For remote screen-based eye trackers, the following setup constraints remain important. Remote screen-based eye trackers offering sampling rates between 60 and 120 Hz are typically sufficient for educational applications, balancing precision, robustness, and practicality. Microsaccades and very small saccades typically require higher sampling rates and low-noise tracking, and may exceed the needs of routine school-based assessments. Higher sampling frequencies may enhance the capture of fine-grained saccade latencies, but they do involve increased costs and more complex setup requirements [15,16]. Key hardware considerations include the following: a stable head position, preferably maintained at a distance of 55–65 cm from the monitor, to facilitate consistent tracking [15]; screen resolution that is adequate to clearly display text and numerical grids, which is vital for minimizing visual distractions [15]; and minimization of reflections on both glasses and screens, as well as controlling ambient lighting to maximize tracking quality throughout the session [15]. By adhering to these hardware guidelines, researchers can ensure improved tracking performance and data quality.

For wearable head-mounted eye trackers, rigid head stabilization is generally unnecessary; instead, data quality depends on secure fit, slippage management, and the characteristics of the scene camera used to map gaze onto real-world materials. Modern wearable systems may also provide higher-frequency gaze and pupillometry streams (e.g., up to 200 Hz), which can be advantageous when studying attention and cognitive load during naturalistic learning tasks.

### 4.2. Calibration and Validation

In this context, calibration and validation procedures are essential to ensure that the recorded metrics are interpretable and comparable across sessions. Accurate calibration is paramount for reliable eye-tracking data [15,16]. It should be noted, however, that calibration requirements depend on the hardware class. While remote screen-based systems typically require explicit multi-point calibration and validation, some recent wearable head-mounted devices implement calibration-free gaze estimation. In these cases, quality assurance should emphasise short validation routines on known targets, reporting of tracking loss and usable-sample percentage, and transparent reporting of device accuracy as documented in independent or manufacturer-provided evaluations (e.g., lab and in-the-wild accuracy reports). A recommended practice involves a 9-point (3 × 3 grid) calibration procedure followed by validation [15,16], aiming for an angular error of less than approximately 1° for most calibration points. Notably, ~1° is also a commonly used upper-bound to define microsaccades (i.e., very small fixational saccades). If the validation error rises due to participant movement or fatigue, it is advisable to recalibrate rather than continue with potentially compromised data [15,16].

When the hardware and sampling rate allow, quantifying microsaccades and other very small saccades can be clinically informative, as subtle oculomotor abnormalities have been reported in several neurological conditions, including pediatric populations.

### 4.3. Data Flow and Reporting

Beyond acquisition itself, ET-based school applications also require a transparent and reproducible data-processing and reporting workflow. An effective data flow process for ET-based interventions encompasses several key stages: calibration and validation; execution of tasks such as reading, copying, arithmetic, or visual search; data export and cleaning, which involves blink removal and artifact detection; calculation of key metrics, including AOI-specific indicators; comparison of gathered data against baseline measures and age-related benchmarks [15,16]; creation of graphical reports, delineating metrics such as regressions per line, mean saccade amplitude, and time spent in irrelevant AOIs.

When generating reports for educators or family members, it is advisable to emphasize visual trends that are easily interpretable rather than delving into complex technical details. For instance, highlighting reductions in regression rates across sessions or increases in reading rates at consistent accuracy levels can provide clear insights into intervention effectiveness [15,16].

## 5. Functional Assessment Guided by ET

ET can enrich school-age assessment by adding a process-level description of how students sample task-relevant information during curriculum activities. In this view, ET does not replace standardized academic or neuropsychological testing; rather, it provides converging evidence about where and when processing breaks down during reading, handwriting/copying, and arithmetic. Functional assessment guided by ET is therefore most informative when it uses grade-appropriate materials, defines task-relevant Areas of Interest (AOIs) a priori, links a small set of eye-movement indicators to an explicit hypothesised bottleneck (e.g., unstable left-to-right progression, reduced model–production coordination, chaotic scanning of columns), and interprets gaze data alongside behavioural outcomes (accuracy, error types, comprehension, and fluency) and contextual factors such as fatigue, motivation, and task familiarity [4,5,6,11,15,16].

### 5.1. Reading: Decoding Efficiency, Integration, and Line-Level Control

In text reading, ET is well suited for capturing the stability of progression within and across lines and to provide indirect markers of decoding and integration effort. A minimal reading assessment battery can include short passages of increasing difficulty (matched for grade and typography) and, where relevant, supplementary word/pseudoword lists or rapid naming arrays. Across these materials, a core set of eye-movement measures can be extracted: mean fixation duration, fixation count per word, forward saccade amplitude, regressions (overall and per line), and indices of line transitions (return-sweep accuracy, line skips, and inter-line saccades) [4,5,6,9,11].

Interpretation should remain mechanism-based and cautious. Longer and more variable fixations, shorter saccades, and increased regressions are commonly observed in struggling readers and in many profiles of SLD, but these patterns are best understood as reflecting inefficiencies in decoding, lexical access, integration, and executive control rather than purely “oculomotor deficits” [12,13,14,15,16]. Regressions, in particular, can serve different functions (e.g., targeted re-reading vs. disorganised backtracking) and should be interpreted in relation to comprehension accuracy, error types, and text difficulty. Accordingly, rather than focusing on a single metric, assessment should examine whether a student’s gaze profile changes systematically as linguistic load increases (e.g., disproportionate growth of regressions and refixations when moving from easier to harder passages), which may indicate fragile automatisation and reduced resilience under demand [11,15,16].

Where hardware and experimental control allow, pupil-based indices can be added as complementary markers of mental effort and sustained engagement, provided that luminance and screen characteristics are controlled and interpreted appropriately [17,18,19]. In applied school reports, however, emphasis should remain on robust, interpretable indicators that map onto observable reading behaviours (e.g., slowed rate with stable accuracy, line-tracking errors, frequent re-reading of specific segments).

### 5.2. Handwriting and Copying: Model–Production Coordination

In handwriting and copying, ET enables a functional characterization of how students coordinate visual inspection of a model (printed or screen-based) with ongoing production. A practical protocol uses materials spanning letters, syllables, words, and short sentences, with AOIs defined for the model region and the writing surface (paper or tablet). Key indicators include initiation latency (time from stimulus onset to first model inspection/production onset), the stability and frequency of fixations on the model, the number and timing of transitions between model and production AOIs, and the latency between the last model fixation and resumption of writing [34].

These measures help distinguish different functional profiles. Some students show over-reliance on the model, with excessive model checking and slow production; others show under-checking or prolonged off-model periods, which can be associated with spatial misalignments, omissions, letter-form errors, or drifting away from the reference. Importantly, such patterns should be interpreted alongside behavioural outputs (legibility, spacing/alignment, omissions, and writing speed) and task constraints (copying vs. composition, length, and familiarity). Within the broader framework of this review, these assessment indicators provide the operational basis for deciding whether a model–production gaze-contingent support is warranted and which outcomes should be prioritised as proximal markers of mechanism-consistent change (e.g., more timely re-checking) versus distal educational outcomes (e.g., improved written products) [34].

### 5.3. Arithmetic: Stepwise Strategy Execution and Visuospatial Alignment

For arithmetic tasks, especially multi-digit, column-based operations, ET can reveal whether students implement a consistent stepwise strategy and whether errors arise from inefficient visual sampling rather than from conceptual misunderstandings alone. A functional protocol can present problems in a grid format and define AOIs for operator position, current column, carry/borrow regions, and any reference cues. Relevant gaze measures include dwell time in target vs. non-target cells, premature AOI exits (leaving a column before adequate inspection), scanpath consistency across trials, and the timing of looks to operators and carry/borrow markers relative to response steps [15,16].

These indicators are particularly useful for identifying visuospatial components of performance (e.g., misalignment, place-value confusions, carry/borrow placement errors) and for informing whether structured scanning supports are plausible mechanisms to test. As with reading and handwriting, interpretation should be anchored to behavioural outcomes: accuracy, latency, error taxonomy, and rule adherence. Where tasks involve number-line estimation, additional measures can quantify gaze efficiency on the number line (e.g., time spent searching, number of corrective saccades, and transitions between anchors and target positions), supporting the assessment rationale for progressive cue-fading approaches [15,16,35,36].

### 5.4. When Attentional Difficulties Co-Occur: Distinguishing Domain-Specific from Domain-General Constraints

SLD frequently co-occurs with attentional and executive-control weaknesses. In these cases, inefficient gaze patterns during academic tasks may reflect either domain-specific difficulty (e.g., decoding or magnitude mapping) or domain-general instability (distractibility and reduced goal maintenance). To help differentiate these pathways, functional assessment can include brief, low-burden tasks such as visual search or short continuous-performance-like blocks, focusing on indicators such as gaze dispersion, time spent off-task AOIs, adherence to task-relevant AOIs, and variability in pupil-based effort (when available) [17,18,19,35,37]. These measures are not intended to diagnose ADHD or other conditions; rather, they support the interpretation of academic task performance and help decide whether preliminary supports targeting sustained engagement should be embedded before domain-specific interventions.

### 5.5. Practical Recommendations for a School-Oriented Assessment Battery

For feasibility in school contexts, we recommend a compact battery that can be completed within a short session and repeated to monitor change: a brief reading block (passages of graded difficulty plus comprehension probes), a copying/handwriting block with clearly defined model vs. production AOIs, and an arithmetic block targeting the relevant curricular procedure (e.g., column operations and/or number-line estimation). Across tasks, the assessment should pre-specify a small set of primary gaze indicators linked to the hypothesised bottleneck (e.g., regressions per line and line-transition errors in reading; model-production transition timing in copying; AOI dwell and scanpath consistency in arithmetic) and pair them with curriculum-relevant behavioural outcomes [11,15,16,34].

ET results should be reported as complementary functional evidence rather than as standalone diagnostic markers. The most informative outputs for educators and families are typically those that connect gaze patterns to observable task behaviours, show within-student change across difficulty levels or across sessions, and remain robust to measurement noise by including data-quality indicators (tracking loss, usable-sample percentage, calibration/validation information when relevant) [15,16,22,32].

School deployment requires minimising burden (short blocks, breaks, and child-friendly calibration/validation) and protecting privacy, especially when wearable systems record scene video. Protocols should specify consent procedures, data minimisation (e.g., storing derived gaze features rather than raw video when possible), secure storage, and clear communication that ET outputs are supportive functional indicators rather than diagnostic labels.

### 5.6. Heterogeneity, Developmental Stage, and Comorbidity in Functional Assessment

Functional assessment should avoid treating specific learning disorders as a uniform category. The same behavioural difficulty may arise from different processing constraints, and similar gaze patterns may reflect different underlying mechanisms depending on developmental stage and comorbidity. For example, elevated regressions in reading may indicate fragile decoding in one student, but disrupted sustained attention or inefficient monitoring in another. Likewise, prolonged dwell times in arithmetic may reflect careful strategy use, confusion about procedural steps, or delayed reorientation after distraction.

For this reason, ET-guided assessment should be interpreted at the level of individual functional profiles rather than as a search for a single disorder-specific signature. At a minimum, reports should specify age/grade, task characteristics, and severity level where available and whether attentional or executive-control difficulties are present. Where the sample size permits, analyses should distinguish developmental subgroups and comorbidity profiles, because these factors are central to the interpretation of gaze metrics in school-age specific learning disorders.

## 6. Gaze-Contingent Intervention Protocols for School-Age SLD

In this paper, “gaze-contingent” refers to closed-loop rules where stimulus visibility, pacing, or feedback is triggered by the learner’s moment-by-moment gaze relative to predefined AOIs. This differs from open-loop supports (e.g., static highlighting, fixed pacing), where adaptation does not depend on gaze. The six paradigms proposed below should be interpreted explicitly as preliminary, hypothesis-driven models for future experimental testing in school-age specific learning disorders, not as established or clinically validated interventions. Their value at present lies in providing a structured framework for mechanism-based study design, outcome selection, and controlled evaluation.

Building on the general principles of gaze-contingent training introduced earlier (i.e., online adaptation of stimuli and feedback based on gaze behaviour), this section focuses on school-age specific learning disorders (SLDs) and summarises SLD-relevant, task-embedded protocols organised by academic domain. Given that controlled trials in SLD are still limited, we distinguish between approaches that are already supported by related evidence and hypothesis-driven paradigms that require formal testing in SLD samples. In this section, we propose six hypothesis-driven gaze-contingent training paradigms (H1–H6), each stated as a testable intervention hypothesis and organised by academic domain (reading, handwriting, and arithmetic).

Accordingly, the rationale for H1–H6 is literature-informed but unevenly supported across domains. Reading-oriented paradigms currently have the strongest indirect empirical grounding, whereas handwriting- and arithmetic-oriented paradigms remain more exploratory. The aim of presenting them is therefore not to recommend immediate routine implementation but to clarify which mechanisms, task rules, and outcome domains future trials should test explicitly.

To clarify the current evidence base and distinguish hypothesis-driven proposals from more established intervention logic, Table 4 provides a compact overview of the six proposed paradigms (H1–H6). For each paradigm, the table summarises the core rationale, the current degree of literature support, the main limitations of the available evidence, and the priority outcome domains for future controlled studies. Because the underlying evidence differs substantially across domains, the table is intended not as a list of validated recommendations, but as a structured map of testable proposals with different levels of empirical grounding.

Compared with open-loop supports (e.g., static highlighting, fixed pacing, or generic computer-based exercises), gaze-contingent delivery enables closed-loop, real-time adaptation based on the learner’s ongoing information sampling. Cues can be triggered only when gaze reaches (or fails to reach) task-relevant AOIs, pacing can be adjusted to engagement, and irrelevant regions can be attenuated contingent on gaze. This supports individualized scaffolding while also enabling mechanistic testing because the manipulation is explicitly tied to the sampled information [7,8,9,10,15,16]. Importantly, this approach generalizes across learning disorders only when the hypothesized bottleneck can be operationalized as inefficient allocation of gaze to domain-relevant AOIs (e.g., word/line progression in reading, operator/column monitoring in arithmetic, or off-task dispersion in comorbid attention difficulties) and when outcomes demonstrate retention/transfer beyond the training display.

Across the six paradigms, gaze-contingent rules are applied to curriculum-relevant materials rather than abstract laboratory stimuli. For reading (H1–H3), tasks typically involve aloud or silent reading of short, grade-appropriate passages (and, where relevant, word/pseudoword lists or rapid naming arrays), presented with controlled typography (font size, spacing, and line length) and with difficulty titrated across sessions. For handwriting/copying (H4), the model can be printed or screen-based (letters, syllables, words, and sentences), with the writing surface (paper/tablet) kept stable and AOIs defined for model vs. production areas. For arithmetic (H5–H6), stimuli should reflect the trained procedure (e.g., multi-digit column operations with carries/borrows; number-line estimation problems), with explicit operator/column AOIs. In addition, we recommend a brief oculomotor screening block (e.g., pro-/antisaccade and basic vergence/binocular checks) to characterize inhibition and binocular constraints that may moderate responsiveness to gaze-contingent reading supports [23,24,25,26,27].

In this context, support was considered direct when the cited evidence involved quantitative intervention data in school-age SLD using the same or a closely comparable training logic, indirect when the rationale was supported by adjacent ET/intervention literature or nearby populations, and primarily theoretical when the proposal rested mainly on mechanistic inference rather than directly relevant intervention data.

As shown in Table 4, the strongest rationale currently exists for reading-oriented paradigms that build on established models of oculomotor guidance and attentional allocation, whereas arithmetic- and handwriting-oriented paradigms remain more weakly supported and should presently be regarded as structured hypotheses for experimental testing. Across all six paradigms, the key interpretive point is that gaze change alone is insufficient: studies should include behavioural outcomes aligned with the relevant academic domain, together with retention and transfer probes under standard, non-contingent conditions.

H1 (Reading—Moving Window): A gaze-contingent moving-window display (maximal clarity/contrast within a window centred on fixation; peripheral attenuation) will reduce distractor-driven scanning and stabilise left-to-right progression in struggling readers, leading to fewer regressions per line and more regular saccade amplitudes during text reading [38,39].

H2 (Reading—Line Guidance/Reading Ruler): A gaze- or position-contingent reading ruler/line guide will reduce line skipping and improve line tracking, reflected by fewer inter-line saccades, fewer return sweeps to incorrect lines, and improved reading rates at comparable accuracy [11,40].

H3 (Reading—Pacing and Analytic Cueing): Gaze-contingent pacing (e.g., advancing to the next segment only after sufficient fixation on the current segment) and dwell-triggered analytic cues (e.g., syllable/grapheme highlighting activated only after prolonged fixations) will promote temporal regularity and reduce impulsive skipping, improving comprehension and decoding efficiency while decreasing excessive refixations on difficult words [35,37,41].

H4 (Handwriting—Model–Production Coordination): In copying/handwriting tasks, gaze-contingent feedback that prompts re-checking the model when gaze remains off-model beyond a threshold will improve model–production coordination, reducing omissions and spatial misalignments and normalising transitions between model and writing area [34].

H5 (Arithmetic—AOI-gated stepwise scanning): In column-based arithmetic, gaze-contingent “AOI-gated” progression (proceeding only after adequate fixation on operator/current column) will enforce stepwise strategies and discourage chaotic scanning, yielding fewer alignment errors and reduced dwell time in irrelevant regions. This hypothesis is consistent with the notion that structured visual-attention training can support academic performance [41,42], and it should be evaluated using domain-relevant gaze markers (e.g., time in non-target cells; scanpath consistency) alongside accuracy indices [15,16].

H6 (Arithmetic—Progressive Number-Line Support): A progressively faded number-line support (from dense cues to sparse cues) will strengthen spatial representation of magnitude and improve strategy use in mental arithmetic [35,36], with expected reductions in inefficient search behaviour and corrective saccades and improvements in both performance and gaze-based efficiency indicators [15,16].

Each hypothesis is expanded in Section 6.1, Section 6.2 and Section 6.3, where we specify candidate outcome measures and practical task parameters.

### 6.1. Dyslexia: Gaze-Contingent Support During Reading

Gaze-contingent reading interventions aim to stabilise oculomotor behaviour and to reduce behaviours associated with decoding effort (e.g., excessive regressions, unstable fixation patterns). One hypothesis-driven approach is a moving-window paradigm, where only a limited region of text around the current fixation is shown with maximal clarity/contrast, while peripheral text is attenuated. This can encourage attentional anchoring to the intended word/region and can be progressively relaxed as reading control improves [39,43].

A second family of strategies targets line tracking and forward scanning. Digital reading rulers (gaze- or position-contingent) can reduce line skipping and help maintain a regular left-to-right progression, potentially reducing disruption from misaligned visual input [11,40]. A complementary approach uses pacing cues (visual and/or auditory) to stabilise the rhythm of fixations and saccades; multisensory pacing has been reported to support attentional control and reading performance in dyslexic individuals [35,37,41].

Contingent cueing for analytic processing can be implemented via syllable or grapheme highlighting that activates only after prolonged fixations (i.e., a “slow-down when needed” rule). The rationale is to promote deliberate grapheme–phoneme mapping and reduce impulsive skipping, while leaving fluent segments unaffected. This approach is best framed as a targeted support for specific difficulty profiles and should be evaluated with both eye-movement outcomes and standardised reading measures [35,37,41].

### 6.2. Dysgraphia: Visual Monitoring and Model–Production Coordination

For dysgraphia (and related handwriting difficulties), gaze-contingent protocols should support the coordination between a visual model (on screen or paper) and ongoing production. A practical paradigm presents a model (e.g., letter string or short sentence) while the student writes; the system provides brief feedback if gaze remains off-model beyond a pre-set duration, encouraging systematic checking of spacing, letter forms, and alignment. Eye-tracking metrics such as transitions between model and writing surface, initiation latency, and model-fixation stability can be used to adapt task difficulty and to quantify change over sessions. Evidence indicates that eye-movement patterns relate to handwriting production and that enhanced visual monitoring can support more accurate execution in children with handwriting difficulties [34]. However, dedicated controlled studies testing gaze-contingent training effects specifically in dysgraphia remain limited, so protocols should be introduced as promising but preliminary and evaluated with clear educational outcomes.

### 6.3. Dyscalculia: Structured Scanning and Alignment in Arithmetic Tasks

This section expands the two arithmetic hypotheses introduced above (H5–H6), specifying candidate gaze-contingent task rules and outcome measures for dyscalculia-oriented training.

#### 6.3.1. H5—AOI-Gated Stepwise Scanning in Column-Based Arithmetic

In column-based arithmetic, a frequent source of errors is inefficient visual sampling (e.g., premature shifts away from the current column, incomplete inspection of operators, or inconsistent scanning across steps). H5 proposes an AOI-gated gaze-contingent paradigm in which progression to the next step is enabled only after sufficient fixation on predefined task-relevant areas (e.g., operator, current column, carry/borrow region). The aim is to promote a stepwise strategy and discourage chaotic scanning, thereby reducing misalignment and procedural errors. This approach is consistent with the broader notion that structured attentional training can support academic performance [38,41].

Evaluation should combine performance indices (e.g., alignment errors, carry/borrow placement, rule adherence, accuracy, and latency) with gaze-based markers such as dwell time in non-target cells, premature AOI exits, and scanpath consistency across trials [15,16]. Support levels (e.g., AOI size, gating thresholds, or the presence of spatial cues) can be progressively faded as students achieve stable performance.

#### 6.3.2. H6—Progressive Number-Line Support with Faded Cues

Difficulties in numerical magnitude representation and spatial mapping are frequently implicated in dyscalculia and related arithmetic impairments. H6 proposes a gaze-supported, progressively faded number-line training paradigm in which initial sessions provide dense spatial cues (e.g., fully marked scales and explicit anchors), followed by a gradual reduction of cues (sparser markings and fewer anchors) to strengthen internal magnitude mapping and strategy use [35,36]. Gaze can be used to trigger minimal prompts (e.g., highlighting anchors only after prolonged search) or to ensure adequate inspection of reference points before responding.

Outcome evaluation should include both behavioural measures (accuracy, response time, error types, and strategy indicators) and gaze-efficiency indicators, such as reduced search time on the line, fewer corrective saccades, and more direct gaze transitions between anchors and target positions [15,16]. As with H5, the intervention should be framed as hypothesis-driven and tested with appropriate controls to determine whether improvements generalise to curriculum-relevant arithmetic tasks.

Across H5–H6, we recommend reporting both curriculum-relevant performance indices (accuracy, error types, and rule adherence) and gaze-based efficiency markers (AOI dwell patterns and scanpath consistency) and embedding these paradigms within the minimal session template described in Section 6.5.

### 6.4. When Attentional Weaknesses Co-Occur with SLD

When attentional control difficulties co-occur with dyslexia/dysgraphia/dyscalculia, short gaze-contingent modules can be embedded to strengthen basic oculomotor persistence and adherence to task-relevant AOIs. Examples include brief smooth-pursuit or visual-search games that introduce a mild cost for leaving relevant AOIs prematurely, with the goal of supporting sustained focus before domain-specific (reading/writing/math) training blocks [38,41]. These modules should remain supportive (not punitive), short, and tightly connected to transfer tasks to avoid overburdening students with comorbidities.

SLD frequently co-occurs with attentional disorders or broader executive-control weaknesses. In this context, an ET signature (e.g., increased regressions or disorganised scanpaths) may reflect either domain-specific processing difficulty (decoding, handwriting planning, and number-processing) or domain-general attentional instability (poor sustained attention and distractibility). To differentiate these pathways, we recommend reporting both domain-specific gaze markers (e.g., word-level regressions; model–production alternations; column-specific dwell) and domain-general indicators (e.g., gaze dispersion, off-task AOI time, variability of pupil-based effort) and, where feasible, stratifying analyses by comorbidity status (e.g., SLD-only vs. SLD + ADHD) [35,37,44].

### 6.5. Minimal Session Template and Reporting of Outcomes

A feasible school-oriented session can be organised as follows (≈30 min): device setup and calibration if required by the system; short baseline task(s) matched to the target domain (reading passage; copying/handwriting sample; arithmetic grid); one or two brief gaze-contingent training blocks separated by micro-breaks; brief post-block probe task to test near transfer [15,16]. Where available, additional indicators such as in situ pupillometry can be used to monitor sustained effort and to help interpret performance fluctuations across blocks.

Progress should be reported using converging indicators, not a single eye-tracking metric. For reading, a credible improvement profile may include reduced regressions per line, increased mean saccade amplitude, shorter/more stable fixations, increased words-per-minute without loss of accuracy, and improved comprehension scores [11]. For handwriting, relevant indicators include improved model-fixation stability, reduced latency between model consultation and writing resumption, fewer omissions, and better spatial alignment/graphic quality [34]. For arithmetic, improvements may include fewer alignment errors, reduced time in non-target cells, and more consistent scan paths supporting correct procedural steps [15,16].

The protocol families outlined above also raise a broader reporting question: what kinds of quantitative change should be considered meaningful in school-oriented ET studies? To help contextualise this issue, Table 5 presents brief illustrative examples of intervention studies from adjacent learning- and attention-related populations that reported pre–post-changes in both behavioural and eye-movement outcomes. The table is not intended as direct evidence that gaze-contingent interventions are already established in school-age SLD. Rather, it shows the types of quantitative outcomes, effect patterns, and training doses that have been reported in nearby areas and thus provides a reporting-oriented bridge between the hypothesis-driven paradigms proposed in Table 4 and the limited empirical literature that has used ET to quantify intervention-related change.

Because existing studies differ substantially in design (open-loop vs. contingent), target domain, training dose, and follow-up, a pooled meta-analytic effect size is beyond the scope of this narrative review. Instead, the goal of Table 5 is to make the quantitative reporting practices explicit (e.g., pre-post means, group × time effects) and to highlight which gaze metrics have been sensitive to short interventions, thereby informing future controlled trials in school-age SLD. Studies from adjacent domains, such as ADHD-related visuomotor or attention training, were included only when they offered child-based quantitative ET examples relevant to process-level change, reporting structure, or interpretation of gaze-sensitive outcomes. Table 3, Table 4 and Table 5 can be read as a structured progression from task-relevant metrics to mechanism-based intervention hypotheses and, finally, to the outcome profiles that future controlled studies should quantify explicitly.

**Table 5:** Illustrative intervention studies from adjacent domains reporting behavioural and eye-movement outcomes.

Study (Ref)	Sample (Age)	Training Dose and Task	Quantitative Outcomes (Examples)
[41]	Children with reading disorder/dyslexia; *n* = 50 (G1 = 25 trained; G2 = 25 control); groups matched for IQ/sex/age	10 min visual attentional training (oculomotor saccade/pursuit exercises + visual search tasks); pre–post-reading task with ET	G1 showed faster reading and shorter fixations at post-test. Total reading time (Text 1) decreased from 59.4 ± 5.0 s to 45.2 ± 5.7 s; fixation duration decreased from 465 ± 25 ms to 411 ± 21 ms. The control group showed no comparable change.
[43]	Children with ADHD; *n* = 30 (G1 = 15 trained; G2 = 15 non-trained); mean age ~8.7 years	10 min visuopostural training (visual search while standing on unstable platform); pre–post-ET + postural assessment	The trained group showed improved oculomotor performance. Pursuit gain increased from 0.83 ± 0.03 to 0.86 ± 0.03, whereas the non-trained group changed from 0.86 ± 0.03 to 0.85 ± 0.03. A significant group × time effect was reported for catch-up saccades (F(1,28) = 11.80, *p* < 0.002, η^2^ = 0.10).

Notes. This table summarises illustrative intervention studies reporting quantitative pre–post changes in both behavioural and eye-movement outcomes. In most cases, eye tracking was used primarily as an outcome measure rather than as a closed-loop training mechanism. Reported values should therefore be interpreted as adjacent quantitative evidence only. Cross-study comparisons should be made cautiously because tasks, metrics, and follow-up procedures differ substantially.

As Table 5 shows, the currently available quantitative evidence is still sparse and methodologically heterogeneous. Nonetheless, it indicates that ET can provide sensitive process-level markers of change alongside behavioural outcomes, especially when studies report both proximal gaze measures and distal task-relevant performance indices. For this reason, future school-age SLD studies should report not only immediate gaze adaptation but also curriculum-relevant outcomes, transfer under standard display conditions, and—where feasible—follow up data.

Two points emerge from the limited quantitative evidence summarised in Table 5. First, measurable changes in oculomotor indices can occur even after brief training exposures, supporting the role of eye movements as sensitive markers of process-level change. Second, the available studies typically involve short-term pre–post-contrasts with limited information on maintenance, generalisation, and transfer to untrained, curriculum-relevant tasks. This matters because eye-movement patterns can be shaped by task constraints and feedback rules; therefore, improvements in gaze metrics should be interpreted as mechanism-consistent only when they co-occur with educationally meaningful behavioural gains and persist beyond the training display. These considerations motivate the reporting and design recommendations discussed next.

**Figure 1:**
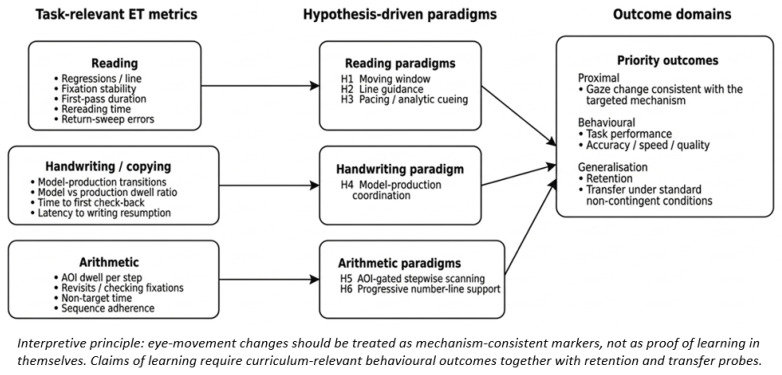
Conceptual summary linking eye-movement metrics, hypothesis-driven gaze-contingent paradigms, and outcome domains in school-age SLD.

To make the internal logic of this review more explicit, Figure 1 summarises the progression from task-relevant ET metrics to hypothesis-driven gaze-contingent paradigms and, finally, to the outcome domains that future controlled studies should prioritise.

Figure 1 shows how domain-specific ET indicators in reading, handwriting/copying, and arithmetic inform the rationale for the proposed paradigms (H1–H6), which in turn should be evaluated using both proximal gaze-based outcomes and distal curriculum-relevant behavioural measures. Across all paradigms, claims of learning should be supported not only by mechanism-consistent changes in eye-movement patterns but also by retention and transfer under standard, non-contingent conditions. H1–H6 should be read as a graded set of experimental proposals. Their current status is not equivalent: Some are adjacent to an existing intervention logic, whereas others remain largely theoretical. In all cases, efficacy claims should depend on controlled testing, curriculum-relevant behavioural improvement, and evidence of retention and transfer beyond the contingent display.

## 7. Discussion

This narrative review argues for a mechanism-based translation of eye tracking into school-age SLD practice: eye movements as process-tracing indicators during core academic tasks and gaze-contingent rules as experimentally testable supports when the hypothesised bottleneck can be operationalised as inefficient sampling of task-relevant information. Building on this rationale, we summarised key metrics and technical requirements for valid acquisition in educational settings, outlined a compact functional assessment approach, and proposed six hypothesis-driven gaze-contingent paradigms (H1–H6) mapped to the current level of empirical support (Table 4). The discussion below focuses on how to interpret change, what constitutes credible evidence of learning (retention/transfer), and which minimal reporting standards are needed to make ET-based interventions comparable and usable in school contexts.

Across gaze-contingent protocols, an apparent improvement in eye-movement metrics should not be interpreted as evidence of learning unless it co-occurs with meaningful changes in curriculum-relevant outcomes (e.g., reading accuracy/comprehension, handwriting quality, arithmetic accuracy) and shows stability beyond a single session. Because gaze can be directly shaped by task rules, some changes may reflect task adaptation rather than genuine skill acquisition. For this reason, intervention studies and applied implementations should include baseline probes, immediate post-block probes, and (when feasible) short follow-ups to evaluate retention and transfer [21].

A central implication of the present review is that eye-movement findings in specific learning disorders should be interpreted within a framework of heterogeneity rather than uniform deficit. Differences across studies are likely to reflect not only methodological variation but also meaningful variation in learner characteristics, including subtype, severity, linguistic and orthographic context, developmental stage, and comorbidity. This is especially important across the broad school-age range considered here. The functional significance of prolonged fixations, regressions, unstable scanpaths, or AOI dwell patterns may change across development as reading, writing, and arithmetic become more or less automatised. Future studies should therefore avoid collapsing broad age bands when interpreting gaze metrics and should discuss explicitly whether observed effects are compatible with delay, divergence, compensation, or domain-general inefficiency.

Comorbidity is equally important. Co-occurring attentional or executive-control difficulties may amplify off-task gaze dispersion, increase variability, and complicate the interpretation of domain-specific performance. From an applied perspective, this means that eye tracking may be most useful not for defining a single “SLD gaze signature,” but for separating different functional pathways that can lead to similar academic outcomes. This also argues for subgroup reporting, developmental stratification, and cautious interpretation of any intervention-related gaze change.

To support comparability across studies and to make ET-based training interpretable in school-age SLD, a minimal reporting set should include the following: hardware class (remote vs. wearable), sampling rate, and calibration approach; data-quality indicators (tracking loss, usable-sample percentage, and validation/accuracy information when applicable); behavioural outcomes (accuracy, response time, and error types) aligned to the targeted academic domain; and a small set of pre-specified eye-movement markers linked to the training hypothesis (e.g., regressions per line and fixation stability for reading; model–production transitions for handwriting; AOI dwell patterns and scanpath consistency for arithmetic) [15,16,22,32]. Where available, additional streams such as in situ pupillometry can help contextualise performance fluctuations, provided that luminance and other confounds are considered. Given heterogeneity and comorbidity, future trials should report subgroup analyses (e.g., SLD-only vs. SLD + ADHD; baseline gaze instability; binocular/vergence markers where available) to identify moderators of response and to avoid overgeneralising protocol effectiveness [14,17].

For school deployment, ET-based protocols should be feasible within short sessions and compatible with classroom constraints. A pragmatic model is to embed gaze-contingent blocks within routine practice activities, using ET outputs primarily for formative monitoring (tracking engagement and strategy use) rather than high-stakes decisions. When used to inform individualised planning, ET measures should complement—rather than replace—standard educational and clinical assessments [15,16]. In addition, successful implementation in schools would require basic teacher and staff training not only in device handling and calibration/validation procedures but also in the cautious interpretation of ET outputs as supportive functional indicators rather than diagnostic labels. This is especially important when wearable systems or scene-video recording are used, since consent, privacy, data minimisation, and communication with families must be managed transparently and consistently.

Evidence in school-age SLD remains heterogeneous, and as summarised in Table 5, most gaze-contingent proposals currently rest on indirect or primarily theoretical support rather than on multiple controlled trials. Future studies should prioritise the following: adequately powered designs with appropriate comparison conditions; clear separation between “support” effects (immediate performance facilitation) and “training” effects (retention/transfer); and transparent reporting of data quality and missingness. Importantly, many SLD profiles are comorbid with attentional difficulties; therefore, protocols should be evaluated for differential benefit across subgroups and for potential unintended effects (e.g., over-reliance on cues or reduced intrinsic strategy use) [21,35,37].

For these reasons, the most defensible contribution of eye tracking at present is not the promise of ready-to-use intervention packages but the possibility of refining functional assessment, testing mechanistic hypotheses, and improving the precision with which future interventions are designed and evaluated.

## 8. Limitations

Several limitations and open questions need to be acknowledged. One major limitation is that eye-movement patterns alone are not diagnostic in isolation; they must be interpreted alongside broader cognitive, linguistic, and educational assessments within established diagnostic frameworks for neurodevelopmental disorders [1]. Even when eye tracking is used for screening approaches in reading, it should be treated as complementary evidence rather than a standalone diagnostic tool [6,7,44], particularly when attentional difficulties are present [44].

Data quality in eye-tracking studies can be compromised by various factors, including participant fatigue, calibration drift, head motion, and device slippage, highlighting the need for meticulous attention to setup, validation, and the experimental environment [15,16,22,32].

Oculomotor improvements observed during training do not necessarily generalize to everyday school tasks unless explicit connections are designed to facilitate transfer. This is particularly relevant for students with comorbidities (e.g., ADHD) or visuospatial attention deficits, who may require preliminary work aimed at enhancing attention, basic oculomotor control, and self-regulation before effective academic training can occur [35,37,38,42].

While the empirical base supporting specific gaze-contingent protocols is gradually emerging, rigorous controlled trials are still needed. These trials are essential to determine which student profiles benefit most from gaze-contingent training, to optimize the structure and intensity of such training, and to evaluate long-term maintenance and generalization of skills acquired through these interventions. This is especially important because the currently available evidence remains distributed across heterogeneous and partially independent studies, including reading-focused ET studies, broader attention/oculomotor training work, and only a limited number of directly school-oriented applied proposals [21,41].

Lastly, advancements in remote and classroom-friendly eye-tracking setups and in adaptive algorithms that adjust parameters such as window size, pacing, and task difficulty in real time represent promising future directions [15,16,45,46]. Future research may also benefit from exploring non-invasive whole-head pose estimation in combination with gaze tracking, particularly in settings where headsets or additional sensors may be impractical. Recent work has shown that facial and gaze comprehension pipelines can combine head-pose estimation, gaze estimation, and eye-state analysis in a non-restrained and non-invasive framework, potentially expanding the range of feasible classroom-compatible monitoring approaches [47]. At the same time, large-scale school implementation will depend not only on technical validity but also on cost, access, maintenance burden, and the availability of systems that reduce repeated calibration demands. In this respect, lower-burden wearable or calibration-light approaches may prove especially relevant for ecologically valid school use, provided that their accuracy and interpretability are adequately validated. Overall, while such innovations may enhance the scalability and ecological validity of ET-informed interventions, they still require careful validation in educational populations to ensure accuracy, interpretability, practical relevance, and equitable accessibility under real-world conditions [15,16].

## 9. Conclusions

The integration of ET technology within educational and rehabilitative programs may support personalised functional assessment and the development of hypothesis-driven interventions for students with specific learning disorders. At present, the evidence base is stronger for using ET to characterise processing patterns and monitor change than for demonstrating intervention efficacy; therefore, gaze-contingent protocols should be treated as experimental and evaluated in controlled designs before being recommended for routine practice [15,16,21,41].

A pragmatic path forward is to connect each gaze-contingent rule to an explicit mechanism (e.g., reducing distractor-driven scanning, supporting line tracking, or prompting model re-checking) and to test whether gains extend to standardised, curriculum-relevant outcomes with retention and transfer probes [4,5,6,11,40]. Advances in wearable systems and classroom-friendly setups can facilitate ecologically valid studies, but they also require transparent reporting of data quality and calibration/validation procedures [22,32,48]. In this sense, school-age SLD provides a valuable applied context to test mechanistic models of reading and attentional control under ecologically valid conditions while generating the controlled evidence needed to judge intervention effectiveness [4,5,6,21,41].

Eye tracking should currently be regarded not as a source of ready-to-use intervention packages but as a means of refining functional assessment, testing mechanism-based hypotheses, and guiding the development of developmentally sensitive and empirically constrained interventions for school-age specific learning disorders.

## Figures and Tables

**Table 2:** Representative search strings used for the literature search.

Domain	Search String
SLD and eye tracking	(“eye tracking” OR “eye-tracking”) AND (“specific learning disorder” OR dyslexia OR dysgraphia OR dyscalculia)
Reading-related tasks	(“eye tracking” OR “eye-tracking”) AND (dyslexia OR reading difficulties) AND (reading OR decoding OR fluency)
Writing-related tasks	(“eye tracking” OR “eye-tracking”) AND (dysgraphia OR handwriting difficulties) AND (writing OR handwriting OR spelling)
Arithmetic-related tasks	(“eye tracking” OR “eye-tracking”) AND (dyscalculia OR arithmetic difficulties) AND (arithmetic OR mathematics OR number processing)
Intervention-focused studies	(“gaze-contingent” OR “gaze contingent”) AND (training OR intervention) AND (reading OR writing OR arithmetic OR learning disorders)

**Table 3:** Recommended eye-movement metrics for school tasks.

Context/Aim	Primary Metrics	Main Interpretation	Practical Considerations
Reading—decoding/ fluency	Median fixation duration; first-pass gaze duration; regression rate; line-transition errors	Online decoding efficiency and attentional guidance; regressions and refixations reflect monitoring and repair under load	Prefer robust statistics; if calibration is unstable, prioritise line-level/AOI measures; control typography
Reading—comprehension/integration	Total reading time; rereading time; regressions to prior lines/clauses; comprehension accuracy	Discourse integration and error detection/repair	Always pair gaze with comprehension outcomes; report speed–accuracy trade-offs; predefine AOIs where feasible
Attention during academic tasks	Dwell time on task AOIs; off-task episodes; latency to return to target AOI; distractor dwell time	Selective/sustained attention, goal maintenance, distractor reorienting	Use robust AOI/time-based measures; define off-task threshold a priori; report distractor salience and task structure
Handwriting/copying	Model–production transition rate; model vs. production dwell ratio; time to first check-back; latency to writing resumption	Visual monitoring and working-memory demands during model–production coordination	Define AOIs consistently; segment by unit/pause where possible; pair with output quality measures
Arithmetic (multi-digit/ column procedures)	AOI dwell per step; sequence adherence; revisits/checking fixations; time in non-target cells	Stepwise strategy execution, verification, and visuospatial alignment	Align AOIs to procedural steps; report error types and rule adherence; standardise layout
Low sampling rate/wearable constraints	Time in AOI; AOI transitions; coarse fixation duration; usable-sample %/tracking loss	High-level attention allocation and strategy, not fine oculomotor physiology	Avoid fine-grained velocity-based indices; emphasise within-student contrasts and converging behavioural outcomes

Notes. AOI = Area of Interest. First-pass gaze duration refers to fixation time during the initial inspection of a word or AOI before gaze leaves it. Rereading time refers to fixation time on previously inspected segments after re-entry. Regression rate and line-transition errors should be operationally defined according to text layout. Off-task episodes and usable-sample percentage should be reported using pre-specified criteria. Across tasks, gaze measures should be paired with curriculum-relevant behavioural outcomes and summarised using robust statistics where possible.

**Table 4:** Proposed gaze-contingent paradigms and priority outcomes.

Proposed Paradigm	Theoretical/Functional Rationale	Current Literature Support (Examples)	Priority Outcomes for Future Studies
H1. Moving window (reading)	Reduces peripheral distraction and supports stable left-to-right progression during reading.	Indirect: supported by gaze-contingent reading/visual sampling studies and adjacent ET reading evidence; direct controlled SLD trials remain scarce [38,39].	Reading speed/accuracy, comprehension, regressions per line, fixation stability, retention/transfer under standard display.
H2. Line guidance (reading ruler)	Supports return sweeps and inter-line transitions without altering core decoding demands.	Indirect: supported by reading ET evidence on line transitions and line-guidance logic; limited intervention evidence in SLD [11,40].	Line skips, inter-line saccades, return-sweep errors, reading rate/accuracy, transfer to standard reading.
H3. Pacing and analytic cueing	Promotes temporal regularity and attention to difficult segments while limiting impulsive skipping.	Indirect: supported by visual-attentional training and cueing studies relevant to dyslexia/reading [35,37,41].	Refixations, skipped words, decoding errors, comprehension, short-term retention/follow-up.
H4. Model–production coordination (handwriting)	Strengthens coordination between visual model checking and written output.	Mostly theoretical/indirect: informed by handwriting-monitoring studies and copying research [34].	Omissions, spacing/alignment, model-to-paper transitions, writing quality, transfer to classroom writing.
H5. AOI-gated stepwise scanning (arithmetic)	Enforces stepwise inspection of operators and digits, limiting premature shifts.	Primarily theoretical: based on AOI-gating logic and general ET methodology, with limited direct dyscalculia evidence [15,16].	Alignment/carry errors, rule adherence, response time, AOI dwell patterns, transfer to curriculum tasks.
H6. Progressive number-line support	Uses fading spatial cues to strengthen magnitude mapping and strategy internalisation.	Primarily theoretical: indirectly informed by visuospatial attention and number-line training literature [35,36].	Magnitude/strategy measures, arithmetic accuracy/RT, gaze efficiency on number line, follow-up transfer.

Notes. The paradigms listed here are presented as hypothesis-driven proposals rather than validated intervention guidelines. “Current literature support” was classified as direct when quantitative intervention evidence was available in school-age SLD using the same or a highly comparable task/intervention logic; indirect when support derived from adjacent ET studies, related interventions, or nearby populations; and primarily theoretical when the paradigm was mainly derived from mechanistic reasoning with little or no directly relevant intervention evidence. Where available, sample size or quantitative anchors are reported in the main text and in Table 5.

## Data Availability

Data are contained within this article.
